# New insights into the pathogenesis of pseudoxanthoma elasticum and related soft tissue calcification disorders by identifying genetic interactions and modifiers

**DOI:** 10.3389/fgene.2013.00114

**Published:** 2013-06-19

**Authors:** Doris Hendig, Cornelius Knabbe, Christian Götting

**Affiliations:** Institut für Laboratoriums- und Transfusionsmedizin, Herz- und Diabeteszentrum Nordrhein-Westfalen, Universitätsklinik der Ruhr-Universität BochumBad Oeynhausen, Germany

**Keywords:** PXE, rare disease, mineralization, calcification, genetic interaction, genetic modifier

## Abstract

Screening of the adenosine triphosphate binding cassette transporter protein subfamily C member 6 gene (*ABCC6*) in pseudoxanthoma elasticum (PXE) revealed a mutation detection rate of approximately 87%. Although 25% of the unidentified disease alleles underlie deletions/insertions, there remain several PXE patients with no clear genotype. The recent identification of PXE-related diseases and the high intra-familiar and inter-individual clinical variability of PXE led to the assumption that secondary genetic co-factors exist. Here, we summarize current knowledge of the genetics underlying PXE and PXE-related disorders based on human and animal studies. Furthermore, we discuss the role of genetic interactions and modifier genes in PXE and PXE-related diseases characterized by soft tissue calcification.

## INTRODUCTION

Mutations in *ABCC6*, a gene encoding for the ABC transporter protein 6 of subfamily C, formerly known as multidrug resistance-associated protein 6 (MRP6), are the cause of pseudoxanthoma elasticum (PXE; Kool et al., 1999; Bergen et al., 2000; Le Saux et al., 2000; Ringpfeil et al., 2000; Miksch et al., 2005; Schulz et al., 2006). PXE is an autosomal recessive disorder characterized by soft tissue calcification primarily in the skin, Bruch’s membrane in the retina and the vessel walls (Neldner and Struk, 2002). To date, more than 300 – mostly unique – PXE-associated *ABCC6* mutations have been identified (). Despite large epidemiological studies including more than 500 well-characterized PXE patients, no genotype–phenotype correlations have been discovered so far. Moreover, there is a great clinical variability between PXE patients, even within families with more than one person affected. This heterogeneity between patients raised the question of whether factors, e.g., environmental or genetic background, contribute to PXE manifestation, disease progression, and severity. The assumption is supported by the description of PXE-related diseases (Vanakker et al., 2007) as well as clinical overlaps with other rare monogenic syndromes and common cardiovascular disorders (Trip et al., 2002). A recent publication in this special issue on soft tissue mineralization focused on the clinical phenotype of PXE and its parallels to other cardiovascular diseases (Lefthériotis et al., 2013). Here, we summarize the role of *ABCC6* mutations as cause of PXE, and discuss the current knowledge of genetic co-factors (modifiers) and genetic interactions for PXE and related disorders.

## MUTATIONAL ANALYSIS OF THE *ABCC6* GENE

Recently, the PXE candidate gene, *ABCC6 *(MIM #603234), was identified and mutations in this gene were found to cause PXE (exemplarily: Le Saux et al., 2000). The *ABCC6* gene contains 31 exons and encodes a 165 kDa transmembrane transporter of 1503 amino acids. The physiological function of ABCC6 is still unknown. To date, more than 300 causative *ABCC6* mutations have been identified. These include missense, nonsense, and splice site mutations, as well as deletions and insertions. According to current studies, PXE seems to be inherited in an exclusively autosomal recessive mode (Plomp et al., 2004; Miksch et al., 2005; Pfendner et al., 2007). Hence, PXE patients are homozygous or compound heterozygous carriers of *ABCC6* mutations. Most mutations observed in *ABCC6* are unique and the majority of them are located within cytoplasmic domains of ABCC6. The most frequent mutation found in PXE patients is a non-sense mutation in exon 24, p.R1141X (c.3421C>T, rs72653706), which is found in approximately 25% of the European patient population. A larger deletion of exons 23–29 (c.2996_4208del) represents the most common mutation (25%) in American PXE patients. The mutation detection rate lies between 80 and 90%. Consequently, in up to 20% of patients clinically diagnosed with PXE, only one or no *ABCC6* mutations were detected. As a consequence of methodological limitations, small *ABCC6* deletions/insertions in homozygous state could be missed by direct sequencing approaches. Costrop et al. (2010) uncovered about 25% of the missing alleles as deletions of various sizes by performing multiplex ligation-dependent probe amplification (MLPA). These findings emphasize the importance of screening for deletion/insertions in the molecular diagnostics of PXE. Nevertheless, there still remain PXE patients with an incomplete *ABCC6 *genotype. Consequently, other genetic causes or environmental factors may be involved in PXE manifestation.

## GENETIC INTERACTIONS

The recent identification of inherited disorders related to PXE and characterized by soft tissue calcification suggests multiple genetic factors. Vanakker et al. (2007) described a novel PXE-like syndrome (MIM #610842), which is inherited in an autosomal recessive mode, but caused by mutations in the *GGCX* gene, encoding γ-glutamate carboxylase. Patients present with generalized redundant skin folds, a mild retinopathy and a coagulation defect of vitamin K-dependent clotting factors (Vanakker et al., 2007). Similar cases had already been reported in the early 1990s (Le Corvaisier-Pieto et al., 1996). Histological examination of a skin biopsy reveals fragmentation and calcification of mid-dermal elastic fibers identical to PXE. However, ultrastructural analysis by electron microscopy showed differences in elastic fiber mineralization: in PXE-like syndrome, the calcification occurred in the periphery of the elastic fiber rather than in the fiber core. Li et al. (2009a,b) described two different families with combined features of PXE and a vitamin K-dependent coagulation deficiency. In both studies mutational screening of *ABCC6*, *GGCX, * and *VKORC1* revealed two putative pathogenic mutations in *GGCX* in patients presenting with distinct skin lesions and a coagulation factor deficiency. Interestingly, the authors observed similar skin lesions in the mother and her twin sister with no evidence of a coagulation disorder (Li et al., 2009a). Mother and maternal aunt were identified as compound heterozygous carriers of the recurrent *ABCC6* nonsense mutation p.R1141X and a missense mutation in *GGCX *(p.S300F). The authors suggested that the skin phenotype is because of digenic inheritance.

Generalized arterial calcification of infancy (GACI, MIM #208000) is another rare autosomal recessive trait characterized by soft tissue calcification (Rutsch et al., 2003). Mutations in *ENPP1*, encoding for ectonucleotide pyrophosphatase 1, are the primary cause of GACI. Patients present with extensive hydroxyapatite deposition in large and medium-sized arteries, leading to arterial stenosis and visceral ischemia. A recent study reported on a family with two brothers (Le Boulanger et al., 2010). One was suffering from PXE, while the other died at 15 months of age from a condition similar to GACI. Molecular analysis of *ABCC6* and *ENPP1* in this family revealed two pathogenic *ABCC6* mutations in heterozygous state as the primary cause of disease. Pathogenic ENPP1 mutations were not identified except for two common ENPP1 sequence variations. It remains speculative whether these lead to a more severe disease progression. Following on from these results, Nitschke et al. (2012) screened *ABCC6 *and reanalyzed *ENPP1* for pathogenic mutations in a larger cohort of patients with clinically proven GACI (*n* = 92). The authors found 30 patients who did not carry any *ENPP1* mutations. Pathogenic *ABCC6* mutations in homo- or compound-heterozygosity were found as the primary cause for GACI in 15 patients (Nitschke et al., 2012). The fact that mutations in *ENPP1* and *ABCC6* manifest in overlapping clinical phenotypes of GACI and PXE suggests similar metabolic pathways are involved in the pathogenesis.

The significance of genetic interactions is further supported by experimental studies with *Abcc6*^-/-^;*Ggcx*^+^^/^^+^ mice in which the onset of ectopic calcification was delayed, whereas *Abcc6*^-^^/^^-^;*Ggcx*^-/+^ mice presented with accelerated mineralization (Li and Uitto, 2010).

## GENETIC MODIFIERS OF PXE

The variability in the outcome and progression of PXE may include variations in several of the functional pathways involved in the pathophysiology of the disease in addition to the loss of ABCC6 function. Detection of such “modifier genes” may uncover major pathways involved in the pathogenesis and provide targets for therapeutic intervention. Studies with *Abcc6*^-^^/^^-^ mice support the hypothesis of the contribution of other genetic factors in PXE-related calcification since these mice present with different phenotypes (Gorgels et al., 2005; Klement et al., 2005).

### DEFINITION OF MODIFIER GENES

The “genetic co-factor” concept is not new, and was introduced by Haldane as early as 1941 (Haldane, 1941). A primary reason for searching for genetic factors modifying disease is to provide information on the disease course and to improve therapies (if available). Several definitions of so-called “modifier genes” exist in the literature. We define a modifier gene as a gene that if “mutated,” is insufficient on its own to cause disease, but when coupled with another genetic mutation, produces or enhances its pathogenesis. “Modifier genes” have been detected for cystic fibrosis, another monogenic disorder caused by an ABCC transporter defect (*ABCC7 *gene) with variable disease onset and progression (Cutting, 2005; Drumm et al., 2005).

### SELECTION OF CANDIDATE GENES

In PXE several metabolic pathways seem to be affected, e.g., the regulation of biological calcification, extracellular matrix (ECM) remodeling and lipid transport and biosynthesis. Sequence variations in genes regulating these pathways may be involved in the development and clinical course of PXE.

#### Regulation of biological calcification

Ectopic calcification is the result of a complex interplay between stimulating and inhibitory proteins and metabolites. One candidate gene for PXE susceptibility is secreted phosphoprotein 1 (*SPP1*, formerly known as osteopontin). SPP1 fulfils important functions in the regulation of biological calcification and was also found to be a constitutive component of elastic fibers in order to prevent them from calcification (Baccarani-Contri et al., 1994). A study by Aherrahrou et al. described a dramatic up-regulation of SPP1 expression in mice suffering from dystrophic cardiac calcification (DCC, Aherrahrou et al., 2004). The *Abcc6* gene was identified as the potential candidate gene for DCC in mice (Korff et al., 2006). *SPP1* is a predominantly transcriptional regulated gene and the *SPP1* promoter is highly conserved among the human, murine, and porcine genes (Hijiya et al., 1994). Several polymorphisms in *SPP1* were shown to affect SPP1 expression (Giacopelli et al., 2004; D’Alfonso et al., 2005; Hummelshoj et al., 2006). We found that the c.-1748G, the c.155_156insG and the c.244_245insTG alleles appear to be significantly more common in PXE patients (Hendig et al., 2007). The polymorphism c.-155_156insG generates a RUNX2 (runt-related transcription factor 2) binding site closed to a second RUNX2 binding site in the *SPP1* promoter. RUNX2 is an essential transcriptional regulator of osteoblast differentiation and bone formation. Conclusively, we interpreted three *SPP1* promoter polymorphisms and the haplotype combining these disease-associated alleles as a putative genetic risk pattern for PXE.

Fetuin-A, was found to be a major systemic inhibitor of calcification (Jahnen-Dechent et al., 2001). Carriers of fetuin-A genotype 2 have been shown to have the lowest serum fetuin-A concentration (Stenvinkel et al., 2005). Even though we did not observe an association between PXE manifestation and fetuin-A genotype, it could be speculated that fetuin-A genotype 2 is an additional disease-promoting risk factor (Hendig et al., 2006).

Matrix gla protein (MGP) is a calcification inhibitor acting locally. MGP was also found in the circulatory system, where it is part of the so-called calciprotein particles, together with fetuin-A and hydroxyapatite. The importance of MGP in preventing pathological calcification is supported by Mgp-deficient mice, which develop severe arterial calcification (Munroe et al., 1999). Analysis of the *MGP* promoter polymorphism frequencies revealed one *MGP* haplotype to be a potential protective genetic co-factor in PXE (Hendig et al., 2008).

#### ECM-remodeling and oxidative stress

Several studies reported an increased ECM remodeling in skin of PXE patients. In this context increased expression of proteases, a mild oxidative stress, and an altered proteoglycan metabolism was detected. The latter resulted in increased accumulation of proteoglycans in the ECM, and in changes in the structure and composition of urinary glycosaminoglycans (Longas et al., 1986; Maccari et al., 2003; Kornet et al., 2004; Volpi and Maccari, 2005).

Many of the alterations observed in PXE could be explained by oxidative stress, for instance ECM remodeling. We found a correlation between genotype and age of disease onset for polymorphisms in the genes catalase (*CAT*, c.262C>T), superoxide dismutase 2 (*SOD2*, c.47C>T), and glutathione peroxidase* 1* (*GPX1*, c.593C>T), encoding essential antioxidant enzymes (Zarbock et al., 2007). Furthermore, the age of disease onset was inversely correlated with the number of mutated alleles, indicating a cumulative effect on the time of PXE onset.

Elevated production of MMP2 (matrix metallopeptidase 2) in PXE fibroblasts and increased levels of MMP2 and MMP9 in serum from PXE patients were shown (Quaglino et al., 2005; Diekmann et al., 2009). Increased MMP expression may at least partly result from genetic variation. We have previously shown that variations in *MMP2* are a genetic co-factor for PXE (Zarbock et al., 2010).

We propose that xylosyltransferase I (*XYLT*, XT-I), as the initial and most important enzyme in proteoglycan biosynthesis, and XT-II, as a highly homologous protein, might contribute to the increased ECM synthesis rate in PXE. As most XT-I is secreted into the ECM, XT activity was proposed as a diagnostic marker for the determination of enhanced proteoglycan biosynthesis and tissue destruction (Götting et al., 2007). Moreover, elevated XT activity was found in sera from PXE patients, reflecting the higher proteoglycan biosynthesis rate (Götting et al., 2005). We further showed that three sequence variants in the *XYLT2 *gene result in a severe disease course of PXE (Schön et al., 2006).

#### Angiogenesis

Choroidal neovascularization (CNV) in PXE-associated retinopathy is believed to be mediated by the action of vascular endothelial growth factor (VEGF). Intravitreal anti-VEGF therapy with ranibizumab or bevacizumab is beneficial for the treatment of CNV secondary to angioid streaks associated with PXE (Gliem et al., 2013). It seems justified to assume that polymorphisms leading to altered VEGF expression may modify the severity of PXE retinopathy. Five *VEGFA *sequence variants showed significant association with severe retinopathy in PXE (Zarbock et al., 2009). We identified the most significant association for the variant c.-460C>T with an Odds Ratio of 3.8 (95% confidence interval 2.0–7.3, *P*_corrected_ 0.0003). *VEGFA* gene polymorphisms might prove useful as a prognostic marker for development of PXE-associated retinopathy leading to earlier therapeutic intervention in order to prevent loss of central vision.

#### Lipid biosynthesis and metabolism

About 50% in the general population present with dyslipidemia which is a high risk factor for coronary artery disease (CAD). Familial hypercholesterolemia is caused by genetic variations in different genes, including low density lipoprotein receptor (*LDLR*) and the apolipoprotein B (*APOB*). Heterozygous carriers are found with a frequency of 1 in 500. Dyslipidemia is likewise observed in PXE patients and case reports suggested an impact of genetic risk factors of lipid metabolism (*LDLR* mutations), for severe complications such as stroke in PXE (Pisciotta et al., 2010).

### GENETIC MODIFIERS IN Abcc6 DEFICIENT MOUSE STRAINS

Genetic examination of nine different mice strains predisposed to more or less severe soft tissue calcification reveal a common *Abcc6* sequence variant in exon 14 as the primary trigger in four of them (c.1866G>A, p.R621C, rs32756904). All these mice strains are prone to soft tissue calcification, but present with immense phenotypic heterogeneity. However, no modifier genes have been identified so far, even though not the correct candidate genes (Ssp1 instead of Spp1) were evaluated in a recent study (Berndt et al., 2012). Whole genome screening and careful evaluation of putative candidate genes will shed light on the role of genetic factors contributing to soft tissue calcification caused by Abcc6 deficiency. It will be of high interest to gain a deeper insight into the genetic background of these mice strains.

## *ABCC6* VARIANTS AS GENETIC MODIFIER OF OTHER RARE AND COMMON DISEASES

The occurrence of a mild skin phenotype was previously reported in heterozygous carriers of *ABCC6* mutations, although the findings could not be replicated in another study (Martin et al., 2007, 2008; Plomp et al., 2009). By searching for biomarkers in biological fluids of PXE patients, we and others observed that unaffected first-degree relatives mostly display results, e.g., for serum fetuin-A, desmosine, that were different from normal and intermediate between that of the PXE patients and the controls (Annovazzi et al., 2004; Hendig et al., 2006). Most of these individuals had been identified as heterozygous carriers of only one *ABCC6* mutation. Taken together, these results underline the assumption that *ABCC6* mutations on a single allele might determine a mild PXE phenotype. This hypothesis might have great impact considering *ABCC6* as genetic modifier of other rare monogenic disorders (GACI), or common disease (stroke, myocardial infarction, and CAD). Wang et al. (2001) reported an association of the frequent *ABCC6 *p.R1268Q variant (c.3803G>A, rs2238472) with plasma triglyceride and low HDL (high-density lipoprotein) cholesterol. Peloso et al. (2010) replicated these data by identification of an *ABCC6* sequence variant (rs150468; c.3736-334A>C) as a susceptibility allele for low HDL cholesterol. Both studies led to the suggestion that ABCC6 may be an important determinant of plasma lipoproteins.

Few studies investigated the correlation of *ABCC6* mutations with cardiovascular disease and identified the frequent mutation p.R1141X as a strong genetic risk factor for CAD (Trip et al., 2002; Wegman et al., 2005; Köblös et al., 2010). However, a large Danish Study including more than 13,600 cases, presenting with ischemic vascular disease, could not replicate this findings (Hornstrup et al., 2011). In conclusion, the impact of *ABCC6* variants as a genetic risk factor for common cardiovascular diseases remains to be elucidated.

## LIMITATIONS AND PERSPECTIVE

Monogenic diseases such as PXE and GACI represent simple models that teach us many things about the genetic basis of more complex disorders, e.g., arterial calcification (Antonarakis and Beckmann, 2006). Considerable variety in the clinical expression of a monogenic disease might be explained by the effect of other genetic factors that modify the expression of the disease phenotype. Many arguments support the concept of modifier genes and genetic interactions. We suggest that genetic interactions, as one example between *ABCC6* and *ENPP1*, point toward new disease entities which seem to be phenotypically similar to PXE or GACI at first sight but differ in yet unknown features. Genetic modifiers are discussed as concomitant factors contributing to the course of the disease. The search for modifier genes is difficult but worth being performed in view of better knowledge of biological pathways affected by the disease. Moreover, numerous studies have identified such genes in mice. The availability of various mouse strains carrying the same *Abcc6* sequence variation associated with more or less severe soft tissue calcification provides an excellent starting point for functional studies and the investigation of interactions between *ABCC6* and specific modifier genes (Berndt et al., 2012). The recent identification of modifier genes for PXE underscores the importance of the analysis of gene–gene environment interactions in understanding the development of complex phenotypes such as PXE. As genetic modifiers are known to alter the course and expression of disease, their gene products become interesting targets for therapeutic intervention. Discovery of genetic modifiers for example affecting the success of anti-neovascular therapy could be, in the future, a key issue to obtain a personalization of therapy and to avoid unnecessary costs in PXE. Nevertheless, replication studies analyzing the new association of modifier genes in other PXE patient cohorts are essential to determine whether these modifier genes are indeed a genetic risk factor for PXE and related disorders. Moreover, the importance of consistent phenotype measures and complementary study designs cannot be overemphasized. Here, family studies and sibling analysis may help to estimate the contribution of genetic and non-genetic factors.

## Conflict of Interest Statement

The authors declare that the research was conducted in the absence of any commercial or financial relationships that could be construed as a potential conflict of interest.
